# Atomic Resolution Structure of the Oncolytic Parvovirus LuIII by Electron Microscopy and 3D Image Reconstruction

**DOI:** 10.3390/v9110321

**Published:** 2017-10-30

**Authors:** Nikéa Pittman, Adam Misseldine, Lorena Geilen, Sujata Halder, J. Kennon Smith, Justin Kurian, Paul Chipman, Mandy Janssen, Robert Mckenna, Timothy S. Baker, Anthony D’Abramo, Susan Cotmore, Peter Tattersall, Mavis Agbandje-McKenna

**Affiliations:** 1 Department of Biochemistry and Molecular Biology, University of Florida, Gainesville, FL 32611, USA; nikea@ufl.edu (N.P.); adammisseldine@ufl.edu (A.M.); lorenageilen@ufl.edu (L.G.); halder.sujata@gmail.com (S.H.); jkennonsmith@ufl.edu (J.K.S.); justinkurian@ufl.edu (J.K.); pchipman@ufl.edu (P.C.); rmckenna@ufl.edu (R.M.); 2 Center for Structural Biology, The McKnight Brain Institute, University of Florida, Gainesville, FL 32611, USA; 3 Department of Chemistry and Biochemistry and Division of Biological Sciences, University of California-San Diego, San Diego, CA 92093, USA; mjanssen@nanoimagingservices.com (M.J.); tsb@ucsd.edu (T.S.B.); 4 Department of Laboratory Medicine, Yale University Medical School, New Haven, CT 06520, USA; anthony.dabramo@yale.edu (A.D.Jr.); susan.cotmore@yale.edu (S.C.); peter.tattersall@yale.edu (P.T.); 5 Department of Genetics, Yale University Medical School, New Haven, CT 06510, USA

**Keywords:** cryo-electron microscopy, oncolytic virotherapy, *Parvoviridae*, structural virology

## Abstract

LuIII, a protoparvovirus pathogenic to rodents, replicates in human mitotic cells, making it applicable for use to kill cancer cells. This virus group includes H-1 parvovirus (H-1PV) and minute virus of mice (MVM). However, LuIII displays enhanced oncolysis compared to H-1PV and MVM, a phenotype mapped to the major capsid viral protein 2 (VP2). This suggests that within LuIII VP2 are determinants for improved tumor lysis. To investigate this, the structure of the LuIII virus-like-particle was determined using single particle cryo-electron microscopy and image reconstruction to 3.17 Å resolution, and compared to the H-1PV and MVM structures. The LuIII VP2 structure, ordered from residue 37 to 587 (C-terminal), had the conserved VP topology and capsid morphology previously reported for other protoparvoviruses. This includes a core β-barrel and α-helix A, a depression at the icosahedral 2-fold and surrounding the 5-fold axes, and a single protrusion at the 3-fold axes. Comparative analysis identified surface loop differences among LuIII, H-1PV, and MVM at or close to the capsid 2- and 5-fold symmetry axes, and the shoulder of the 3-fold protrusions. The 2-fold differences cluster near the previously identified MVM sialic acid receptor binding pocket, and revealed potential determinants of protoparvovirus tumor tropism.

## 1. Introduction

An increasing number of oncolytic viruses debulk tumors, often with a higher therapeutic index than conventional treatments [1,2,3]. October 2015 marked an important milestone with the first commercial virotherapy, talimogene laherparepvec (T-VEC), approved for cancer treatment in the United States and Europe [4]. Members of the *rodent protoparvovirus I species*, including LuIII, H-1 parvovirus (H-1PV), and minute virus of mice (MVM), are utilized as replication-competent vectors to target cancer [5,6]. All three viruses are pathogenic to rodents, and selectively replicate in and kill human cancer cells [7,8,9]. Importantly, a Phase I/IIa clinical trial has extended these observations to patients in which H-1PV was used to treat recurrent glioblastoma multiforme tumors [10,11,12].

LuIII, along with H-1PV, and MVM, belong to the *Parvoviridae*. These viruses package linear ssDNA genomes of ~5 kb into non-enveloped capsids of ~260 Å in diameter [13]. The rodent protoparvoviruses share the same genome structure of two open reading frames, non-structural (NS), and capsid (CAP). NS encodes for three non-capsid proteins (NS1/2 and SAT), while CAP encodes two capsid viral proteins (VPs, VP1 and VP2). Both ends of the viral genome are capped by palindromic sequences responsible for initiating transcription and packaging viral genomes into preassembled capsids [14,15]. The viruses enter cells via receptor-mediated endocytosis, and are trafficked to the nucleus for progeny production. Parvoviruses rely on the host cellular machinery for DNA replication, but lack the accessory proteins required to push cells into the S-phase of the cell cycle [16]. For the rodent parvoviruses, NS1 regulates the viral replication required for tumor cell lysis, and is activated by host cell factors upregulated in actively dividing, dysregulated cancer cells. The result is an S-phase dependent inherently oncoselective virus that can spread from cell to cell throughout a tumor, but otherwise does not infect non-cancerous human cells [10,17,18].

The T = 1 icosahedral parvovirus capsids consist of 60 VPs, with the protoparvoviruses assembled from VP1 and VP2 in a 1:10 ratio. The overlapping VP1 and VP2 sequences share a common C-terminus, with the unique VP1 N-terminus (VP1u) carrying a phospholipase A2 (PLA2) domain [19]. This enzyme is an absolute requirement for parvovirus infection. Upon cell entry, particles containing viral genomes are proteolytically cleaved at the VP2 N-terminus to produce VP3 that becomes the major capsid protein with the capsids now assembled from VP1:VP2:VP3 in a 1:1:10 ratio [20,21,22,23]. Understanding capsid structure as it relates to critical stages in the viral life cycle can drive improvements in vector development. LuIII kills transformed cell types that are either completely resistant, or less easily infected and lysed by H-1PV and MVM. Studies in two different tumor models have correlated this LuIII phenotype to VP2 [7,24,25]. Studies of H-1PV and other rodent protoparvoviruses also support a tumor tropism determinant within this protein [26]. VP2 forms virus-like-particles (VLPs) for members of the protoparvoviruses that are otherwise antigenically and structurally equivalent to VP1/2/3 genome packaging capsids [27,28,29]. This thus provides a reagent for characterizing the tropism determinants for these viruses.

The structures of H-1PV and MVM are available [28,30]. To enable comparative analysis for identifying the VP2 determinant of selective tumor tropism by the protoparvoviruses, the structure of LuIII VP2 VLPs was determined by cryo-electron microscopy and 3D image reconstruction (cryo-reconstruction) to 3.17 Å resolution. At this resolution, the VP2 amino acid side-chain conformations were unambiguously assigned into the cryo-reconstructed density map, from N-terminal residue 37 to the last C-terminal residue 587 (VP2 numbering). This stretch of amino acids is comparable to those ordered in the H-1PV and MVM crystal structures. The VP2 structure topology conserved the core secondary structure elements reported for other parvoviruses and the single 3-fold protrusion capsid morphology of other non-human protoparvoviruses. A comparison of the LuIII VP2 and capsid structures to H-1PV and MVM identified common features within the core regions, and differences on the capsid surface, localized at or close to the icosahedral 2-fold axes, the top of the 3-fold protrusion, and the channel at the 5-fold axes and its surrounding depression. The 2-fold structural differences cluster around the previously reported MVM sialic acid receptor-binding site, while the 3-fold differences map to an MVM antigenic epitope. Furthermore, sequence and structural features unique to LuIII surround the MVM sialic acid binding site utilized for recognition of the sialylated sLeX glycan motif, Neu5Acα2-3Galβ1-4(Fucα1-3)-GlcNAc, abundant on tumor cell surfaces. Thus, this comparative analysis identified loci specific to the rodent parvoviruses that represent “hot spots” of capsid diversity, and potential determinants of tumor tropism and lytic function.

## 2. Materials and Methods

### 2.1. LuIII VLP Expression and Purification

LuIII VLPs were produced using the baculovirus/*Spodoptera frugiperda* (*Sf9*) cell expression system, as previously reported for other parvoviruses (e.g., [27,31]). A recombinant baculovirus encoding the VP2 of LuIII was generated using the Bac-to-Bac system, per the manufacturer’s instructions (Invitrogen, Carlsbad, CA, USA). This recombinant virus was used to infect *Sf9* cells at a multiplicity of infection of 5. For purification, cells harvested 5–7 days post infection were pelleted and resuspended into 25 mM Tris-HCl, 500 mM NaCl, 0.2% Triton X-100, 8 mM CaCl_2_, 2 mM MgCl_2,_ at pH 8.0 (Buffer A). The resuspended cells were subjected to three freeze/thaw cycles, and purified by sucrose cushion. The supernatant from the harvested cells was precipitated with the addition of PEG8000 (10% *w*/*v*) and 500 mM NaCl, stirring overnight at 4 °C, followed by centrifugation at 9000 rpm on a Beckman Coulter JA-10 rotor for 1.5 h at 4 °C. The resulting pellet was resuspended into Buffer A and combined with the resuspended cell pellet sample. All samples were treated with 100 U/mL Benzonase (Millipore, Burlington, MA, USA) for 30 min at 37 °C, and subjected to ultracentrifugation through a 20% sucrose cushion at 45,000 rpm on a Beckman Coulter 70Ti rotor for 3 h at 4 °C. The resulting pellet was resuspended in the same buffer as above, supplemented with 1 mM EDTA, and further purified using a step gradient containing 5–35% sucrose centrifuged at 35,000 rpm on an SW41Ti Beckman Coulter rotor for 3 h at 4 °C. Fractions collected from the 25–35% sucrose regions were dialyzed into 10 mM Tris-HCl, 500 mM NaCl, 8 mM CaCl_2_, 2 mM MgCl_2_, at pH 7.5 (Buffer B). To achieve the desired concentration for cryo-electron microscopy (cryo-EM) data collection, dialyzed sample was pelleted by ultracentrifugation at 50,000 rpm on a SW55Ti rotor (Beckman Coulter, Brea, CA, USA) for 1 h at 4 °C and resuspended in Buffer B to a final concentration of 0.75 mg/mL. To assess sample purity, VLPs were analyzed by 10% Sodium dodecyl sulfate polyacrylamide gel electrophoresis (SDS-PAGE) stained with GelCode™ Blue (ThermoScientific, Waltham, MA, USA).

### 2.2. Sample Preparation and Cryo-Preservation

To confirm LuIII VLP integrity, the sample was visualized by transmission electron microscopy (EM). Five microliters of purified VLPs at 0.1 mg/mL were applied to a glow discharged 400-mesh carbon-coated copper grid (Electron Microscopy Sciences, Hatfield, PA, USA) for 2 min, and blotted with filter paper to remove excess liquid (Whatman No. 5, GE Healthcare Life Sciences, Marlborough, MA, USA). The grid was washed 3× by floating on deionized water, blotted, stained with 2% uranyl acetate for 30 s, and blotted again prior to visualization. The negatively stained sample was viewed using the Tecnai Spirit microscope (FEI, Hillsboro, OR, USA) operated at 120 V, and images were recorded at a magnification of 42,000× on a 16-megapixel charge-coupled device camera (Gatan, Inc., Pleasanton, CA, USA). Following the confirmation that the VLPs were intact, 3 μL aliquots of sample were pipetted onto glow-discharged copper grids containing 2 nm carbon support over holes (Quantifoil R 2/4 200 mesh, Electron Microscopy Sciences). The grids were blotted and the sample vitrified using a Vitrobot Mark 4 (FEI) operated at 95% humidity and 4 °C.

### 2.3. Cryo-EM Data Collection

Prior to cryo-EM data collection on a high-end microscope, a cryo-preserved LuIII grid was screened in-house to confirm optimal sample quality and ice thickness on a Tecnai G2 F20-TWIN microscope operated under low-dose conditions (200 kV, ~20 e^−^/Å^2^), with images collected on a Gatan UltraScan 4000 CCD camera (Gatan, Inc., Pleasanton, CA, USA). The dataset utilized for the LuIII structure determination was collected using the Leginon application on a Titan Krios electron microscope (FEI) operated at 300 kV [32]. A nominal magnification of 130,000× and a pixel size of 1.1 Å was used. The microscope was equipped with a Gatan post-column imaging filter (GIF) utilizing a slit width of 20 eV. Movies were recorded on a Gatan K2 Summit direct electron detection camera operating under counting mode and an accumulated dose of 75 e^−^ per Å^2^ fractionated into movie stacks of 50 frames per micrograph (see Table 1). This data set was collected as part of the NIH “West/Midwest Consortium for High-Resolution Cryo Electron Microscopy” project.

### 2.4. Movie and Image Preprocessing, and 3D Map Reconstruction

To correct for beam induced motion, the movie frames were aligned using the MotionCor2 application [33]. The dose-weighted images averaged from all 50 frames were used for contrast transfer function (CTF) estimation using the CTFFIND4 application [34]. Particle selection was conducted using the automated particle picking option (M) within the AUTOPP subroutine in the AUTO3DEM application [35]. Particle pre-processing to normalize and apodize the particle images also utilized the AUTOPP subroutine (options F and O) of AUTO3DEM [35]. The structure determination utilized the gold standard protocol in AUTO3DEM [35]. A low resolution (30 Å) initial model was generated from 100 particle images using the *ab initio* model generating subroutine within AUTO3DEM, while applying icosahedral symmetry. This model was used to search the origins and orientations of the entire particle data set followed by cycles of origin and orientation refinement, solvent flattening, and CTF refinement. To minimize the effect of radiation damage on the particle images that could have occurred during data collection, the movie frames were re-aligned while truncating the number of frames to include 2 to 30 only. The particle origin and orientation information from the CTF refinement output were applied to the truncated frame particle images, and used for another several rounds of origin and orientation refinement, followed by B-factor (temperature factor) correction within AUTO3DEM. Different B-factor values, 1/50, 1/80, 1/100, 1/150 and 1/200, were applied to the final map, and visually inspected in the Coot and Chimera programs [36,37]. The B-factor 1/50 corrected map was selected for model building, because it showed the most ordered amino acid side chains, with the minimal amount of background noise. However, the 1/100 and 1/150 B-factor corrected maps were utilized for model building of highly flexible surface loops. The resolution of the reconstruction was estimated to be 3.17 Å based on a Fourier Shell Correlation (FSC) of 0.143.

### 2.5. Model Building and Structure Refinement

A homology model of LuIII VP2 was generated using the SWISS Model online server (https://www.swissmodel.expasy.org/) from the LuIII VP2 sequence (NCBI accession # P36310.2) and the Protein Data Bank (PDB) coordinates of MVM (PDB ID: 1z14) as a template [28,38,39]. The VP2 monomer model was used to generate an all-atom 60mer using the online VIPERdb Oligomer Generator for a T = 1 capsid by icosahedral matrix multiplication (http://viperdb.scripps.edu) [40]. The 60mer was docked into the cryo-reconstructed map using the “Fit-in-map” function in the Chimera program [37]. Prior to this fitting, the density map was converted from the Purdue Image Format (PIF) to an XPlor format using the “e2proc3D.py” subroutine in EMAN2 [41]. Following the fitting, the map was converted to a CCP4 format using the MAPMAN application and resized to a 1.064 voxel size to optimize the correlation coefficient between map and model [42]. A reference VP2 monomer was extracted from the fitted 60mer and the side-chains were adjusted, guided by the LuIII cryo-reconstructed map, by manual building and the real-space-refinement subroutine in the Coot program [36]. Residues 37–587 of VP2 were built into the B-factor corrected maps at a sigma (σ) threshold of >1.0.

The VP2 model was used to generate a full 60mer capsid using the NCS extension in Coot. The capsid was refined with an inverse 50 Å^2^ B-factor corrected map utilizing the rigid body, real space, and B-factor refinement subroutines in the Phenix application [43]. The refinement steps were alternated with model visualization and adjustment in Coot to maintain model geometry as well as rotamer and Ramachandran constraints [36]. The correlation coefficient (CC) and root mean square deviations (RMSD) from ideal bond lengths and angles, as reported in Table 1, were analyzed by the Phenix and Molprobity programs [43,44]. All secondary structure elements were assigned based on phi and psi angles (in Molprobity and Coot) and visual inspection of hydrogen bond distances in Coot [36,44].

### 2.6. Structure Comparison

The available *Protoparvovirus* VP2 3D structures were compared by secondary structure matching (SSM) in PDBeFOLD [45]. LuIII was superposed onto H-1PV, MVM, canine parvovirus (CPV), feline panleukopenia virus (FPV), and porcine parvovirus (PPV) by pairwise residue alignment (PDB IDs: 4g0r, 1z14, 2cas, 1c8f, and 1k3v, respectively) [28,30,46,47,48]. However, SSM does not report a Cα deviation value for non-overlapping atoms. Thus, in cases where an amino acid is offset in aligned structure (i.e., due to insertion or deletion of a residue) the distance measurement tool in the Coot program [36] was used to determine a distance to the nearest Cα atom. Variable regions (VRs) among the *Protoparvovirus* were assigned as previously defined, two or more adjacent residues with Cα atom distances of ≥2.0 Å between the superposed structures [30]. The viral capsid surface topologies were also compared among the rodent protoparvoviruses. VIPERdb [40] 60mers, built from the H-1PV and MVM VP2 structure coordinate files (PDB ID: 4g0r and 1z14 ), were each docked into the LuIII cryo-reconstructed map using the “Fit-in-map” function in the Chimera program [37], and used to generate molecular surface maps (molmaps) filtered to 3.17 Å resolution. These H-1PV and MVM molmaps were compared to the LuIII cryo-reconstructed map by visual inspection in the Chimera program [37].

### 2.7. Figures

Figures 2–5 were prepared using the Chimera program [37]. Two-dimensional (2D) Roadmap (asymmetric unit surface) projections (Figures 6 and 7) were generated by the RIVEM program [49].

### 2.8. Accession Numbers

The cryo-EM reconstructed map and capsid atomic model have been deposited with accession numbers EMD-7071 and 6B9Q, respectively, in the Electron Microscopy Data Bank (EMDB) and the PDB.

## 3. Results and Discussion

### 3.1. Cryo-EM and 3D Image Reconstruction Provides Atomic Resolution Information for LuIII VP2

**Figure 1:**
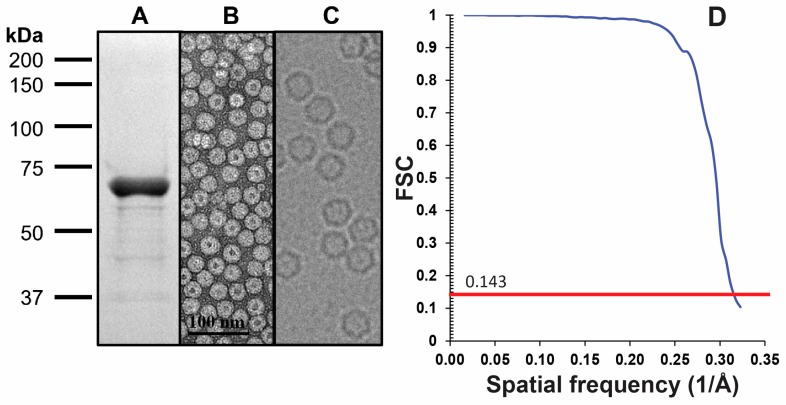
LuIII sample and data quality. (**A**) SDS-PAGE of purified VLPs. The VP2 is ~65 kDa in size; (**B**) negative stain EM of purified VLPs (42,000×, Scale bar = 100 nm); (**C**) representative micrograph from cryo-EM data collection, not visualized to scale; (**D**) FSC plot for the final iteration of 3D map reconstruction (red line indicates the 0.143 threshold utilized to estimate resolution).

**Figure 2:**
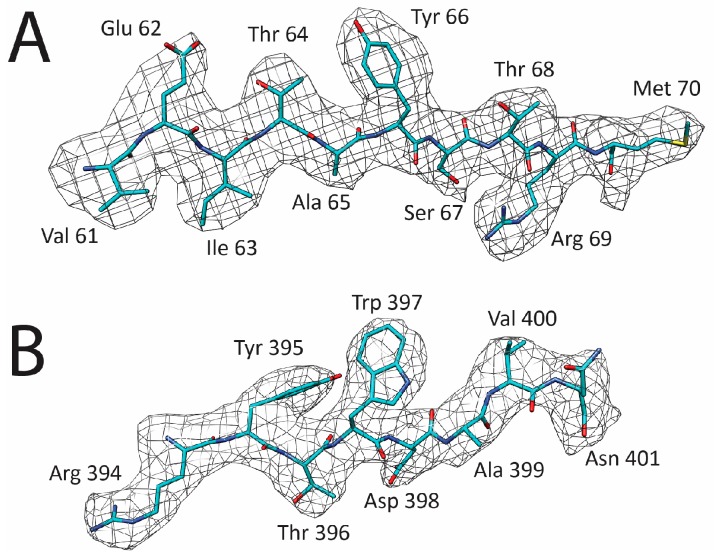
Representative density from the LuIII cryo-reconstruction. (**A**) Region from the βB strand located in the capsid core; (**B**) solvent exposed loop (394–401) on the capsid surface. Map density is in gray mesh (at σ = 1.0) and the LuIII model is in cyan. Residues are as labeled with atoms colored C = cyan, N = blue, O = red, S = yellow.

The capsid structure of LuIII was determined by cryo-reconstruction to 3.17 Å (FSC 0.143) resolution from 18,134 out of 20,142 particle images extracted from 722 micrographs collected for purified VLPs (Figure 1, Table 1). The truncation of initial and latter movie frames moderately improved the map resolution from 3.23 to 3.18 Å, and a reconstruction with 90% of the data set further increased the resolution to 3.16 Å. Inverse B-factor correction resulted in the 3.17 Å resolution map with improved amino acid side-chain density. The cryo-reconstructed map was interpretable for LuIII VP2 N-terminal residue 37 to the last C-terminal residue 587 (Table 1). Density within the VP2 core was interpretable at a σ threshold of 6, while the surface loops were ordered at thresholds of 2 to 4 σ. Example side-chains built into the cryo-reconstructed density are shown in Figure 2. The resolution of the map enabled accurate assignment of ~97% of the 551 residues built for VP2. The final capsid model was refined with a correlation coefficient of 0.88 into the cryo-reconstructed density and the VP2 exhibiting good geometry and Ramachandran statistics (Table 1).

As previously reported for *Parvoviridae* VP crystal structures, the first 37 N-terminal residues of VP2 were disordered [28,30,46,47,48]. Density is visible for the main-chain of 2–4 additional residues at the N-terminus at ≤0.8 σ threshold, but they extend in three different directions. Lack of continuous density in the different paths precluded model building. One conformation extends towards the base of the channel at the icosahedral 5-fold symmetry axis of the capsid, suggesting a possible VP1u externalization path in VP1 containing capsids. LuIII residue 37 is contained within a glycine-rich VP1/VP2/VP3 overlapping region, predicted to allow conformational flexibility that enables VP1u repositioning for externalization for PLA2 function [50]. Thus, the lack of N-terminal ordering is predicted to be due to flexibility, intrinsic disorder, or adoption of alternative conformations by all three overlapping VPs in capsids assembled from VP1, VP2, and VP3.

**Figure 3:**
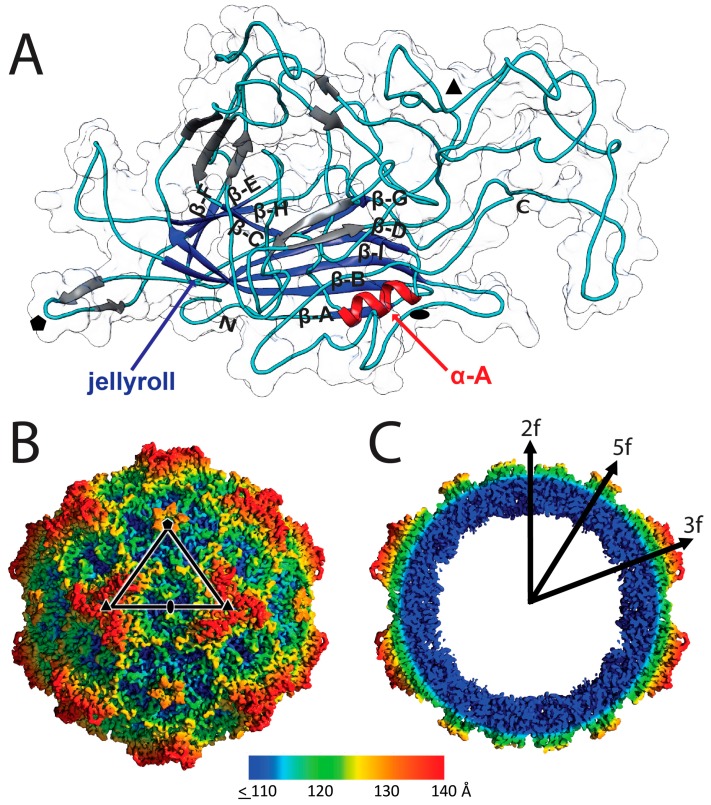
LuIII structure. (**A**) A VP2 monomer, 60 copies of which form the viral capsid. A cartoon is shown overlaid on a transparent surface representation. The jellyroll motif (blue), α-A helix (red), intervening loops (cyan), and short β-strands (gray) are shown. Arrows indicate anti-parallel β-strands with directionality from N- to C-terminus. N = N-terminus (VP2 37) and C = C-terminus (VP2 587). The approximate 2-fold, 3-fold, and 5-fold icosahedral axes are indicated as a filled oval, triangle, and pentagon, respectively; (**B**) surface image of the LuIII cryo-reconstructed map (at σ = 1) radially colored from the capsid center (see color key for distance in Å). The viral asymmetric unit is depicted in the black triangle with white outline. The icosahedral axes are indicated as in (**A**); (**C**) radially colored central section of the cryo-reconstructed map (at σ = 1). The color key is as in (**B**).

A second region of less ordered density occurred at residues 159–164 that form the apex of the β-ribbon, forming the 5-fold channel via interaction with four symmetry related β-ribbons (Figure 3A). However, the main-chain of these six residues were traceable at 1σ. Analogous residues are reported to have high thermal motion (B-factor) or are disordered in other *Parvoviridae* structures, including for H-1PV and MVM [28,30,46,47,48]. Flexibility or disorder of the loop residues forming the apex of the 5-fold channel would enable one of its proposed functions, the portal for externalization of VP1u for PLA2 function.

### 3.2. LuIII Conserves General Parvoviral Structural Features

The LuIII VP2 monomer structure contains the core secondary structure elements reported for other *Parvoviridae* structures, with nine β-strands (βA-βI) and an α-helix (α-A) (Figure 3A). The conserved jellyroll motif contains eight antiparallel β-strands that form two β-sheets (β-BIDG and β-CHEF). Each BIDG β-strand is on average 11 amino acids long, while the β-strands forming CHEF are comparatively shorter with an average of 4 amino acids per strand (Table 2). This results in a jellyroll that is orientated with its open end furthest from the 5-fold axes. Other components of the viral core pack tightly around this motif. Preceding β-BIDG is a short β-A strand that is antiparallel to β-B, and positioned on the small arm connecting β-C to β-D is the α-A helix. The core β-strand and helical regions are connected by loop regions that form the viral surface (named by their position between jellyroll β-strands, for example, the GH loop connects β-strands G and H). A number of short, 2–5 residue long, antiparallel β-strands are interspersed within these connecting loops (Table 2, Figure 3A).

The LuIII capsid is ~280 Å in diameter from 3-fold to 3-fold, similar to previous reports for H-1PV and MVM (Figure 3B,C) [28,30]. The morphology of the parvovirus capsid arises from the interaction of VP surface loops around icosahedral symmetry axes (Figure 3A,B). Five interacting β-ribbons within the DE loop of related VP2 monomers form open channels at each icosahedral 5-fold symmetry axis surrounded by a depression. At the 3-fold symmetry axes, surface protrusions assemble by interacting GH loop residues. Narrow depressions occur along 2-fold symmetry axes. These features are conserved in all protoparvovirus structures determined to date and the 2- and 5-fold features are conserved in all parvovirus capsids. The 3-fold protrusions of parvoviruses vary between a single structure, as observed for the animal protoparvoviruses, or three protrusions surrounding a depression at the 3-fold symmetry axes [51].

### 3.3. Variable Regions Confer Rodent Parvovirus Specific Surface Topologies

**Figure 4:**
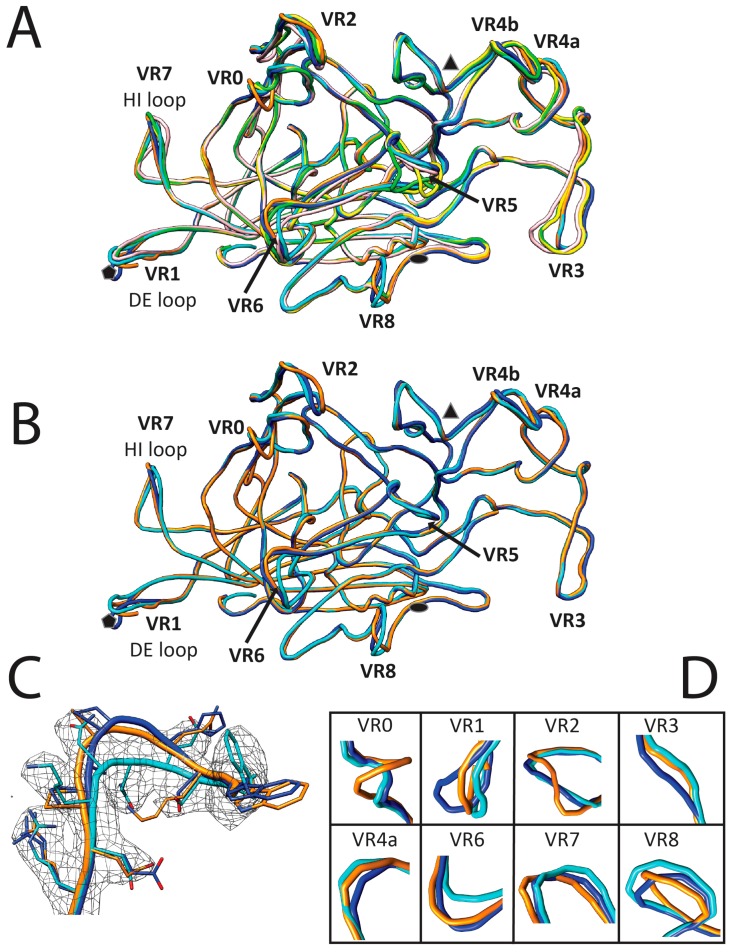
VP2 structural alignment of the protoparvoviruses. (**A**) Superposition of protoparvovirus VP2 structures (shown in cartoon without secondary structures assigned) with variable regions (VRs) labeled; (**B**) superposition of rodent protoparvovirus VP2 structures with VRs labeled; (**C**) LuIII VP2 residues 386–394 of VR6 within the cryo-reconstructed map highlighting ability to distinguish surface loop conformations between the rodent parvoviruses at the resolution of the map. Map density is shown in gray mesh at threshold of σ = 1.0; (**D**) close-up of VRs with the largest differences between the rodent protoparvoviruses. In A LuIII is in cyan, H-1PV in orange, MVM in blue, CPV in green, FPV in yellow, and PPV in pink; in B-C LuIII is in cyan, H-1PV in orange, and MVM in blue. In A and B, the approximate icosahedral 2-fold, 3-fold and 5-fold symmetry axes are indicated as filled oval, triangle, and pentagon, respectively.

Previous comparison of protoparvovirus surface loops have reported “hot spots” of sequence and structural variation, variable regions (VRs), defined as regions with two or more contiguous residues with Cα distances >2.0 Å between the superposed VP structures [28,30,46,47,48]. Secondary structure matching (SSM) used to compare LuIII to the MVM, H-1PV, CPV, FPV, and PPV VP2 structures confirmed the positions of the 10 common VRs for LuIII (Table 3, Figure 4). Compared to each protoparvovirus, the largest LuIII structural variations occur within VR1, VR0, VR5, and VR8 with MVM, H-1PV, CPV/FPV, and PPV, respectively. Overall, the LuIII VP2 is structurally most similar to MVM, closely followed by H-1PV, and is most different from PPV in agreement with amino acid sequence identities (Table 3). Importantly, each VR is located on a surface loop, consistent with structure and antigenic distinction because of sequence diversity within these regions. Predictively, cell attachment and antibody recognition functions utilize residues within the VRs [52,53,54,55,56,57,58,59,60].

**Figure 5:**
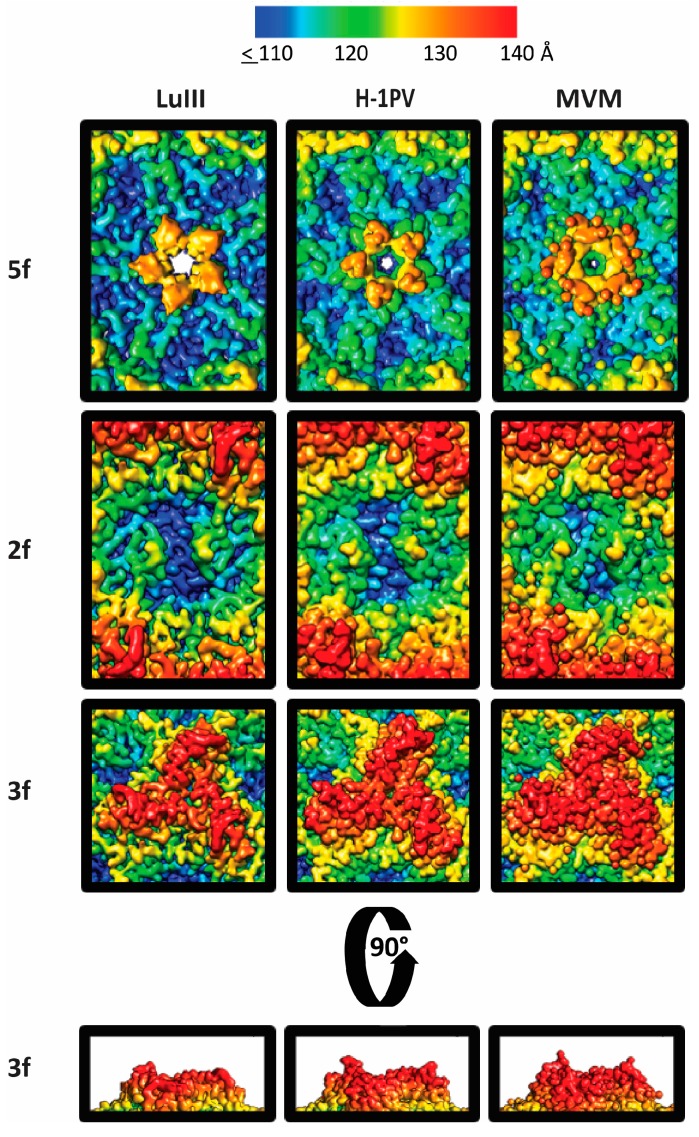
Comparison of surface topology among the rodent protoparvoviruses. Radially colored surface images for the rodent protoparvoviruses (see color key in Å), with close ups of the icosahedral 5-fold, 2-fold, and 3-fold symmetry axes at the top, middle, and bottom, respectively. A 90° rotation is also shown for the 3-fold protrusions. LuIII has an open 5-fold channel surrounded by a depression that is wider, an expanded 2-fold depression, and less pronounced 3f protrusion. The LuIII structure was compared to molecular maps of H-1PV (PDB ID: 4g0r) and MVM (PDB ID: 1z14) docked into the cryo-reconstructed LuIII density map, and filtered to 3.17 Å resolution.

Variable regions create local morphological differences on the LuIII, H-1PV, and MVM capsid with the most pronounced being at the 2-fold axes. In LuIII, this depression is 4–7 Å deeper, and 15–22 Å wider than H-1PV and MVM, respectively (Figure 5), resulting from multiple subtle side-chain differences within residues in VR5, VR6, and VR8. The wall that borders this 2-fold depression (which separates it from the depression surrounding the 5-fold axis), referred to as the 2/5-fold wall, exhibits differences due to the clustering of VR3 andVR6. At the 3-fold axis, the single “windmill” shaped protrusion is the same height in all three viruses, but is narrower in LuIII compared to H-1PV and MVM, and has the same curvature for LuIII and H-1PV (Figure 5). Differences in regions of VR0, VR2, VR3, VR4a, and VR4b contribute to this feature. At the 5-fold region, the depression appears wider in LuIII, due to a difference in the HI loop (VR7) residues that line the floor of the depression, and differential positioning of the apex of the DE loop (VR1) results in a 5-fold channel that is more open at the apex for LuIII (Figure 5). The HI loop is reported to play a role in genome packaging for the dependoparvoviruses [61], while the channel is reported to play a role in VP1u externalization for its PLA2 function, and serves as a portal for genome packaging and uncoating for all parvoviruses [50,62,63].

### 3.4. Variable Regions Confer Tumor Tropism and Cell Killing Efficiency

**Figure 6:**
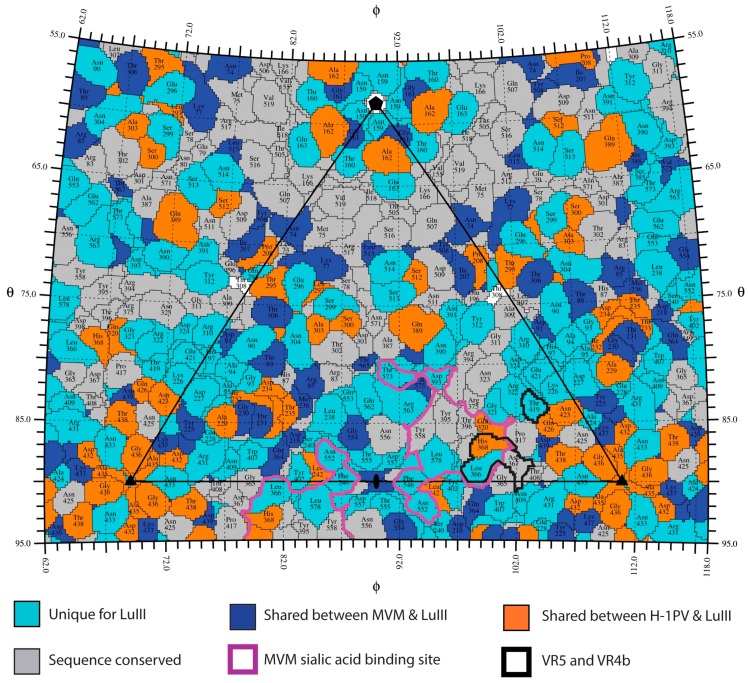
Distribution of amino acid sequence identity for the rodent protoparvoviruses. 2D projection roadmap of the LuIII capsid surface showing the viral asymmetric unit (black triangle). Colors are as indicated.VP2 positions containing an amino acid that is unique to LuIII are in cyan. Residues identical between: MVM and LuIII (blue), H-1PV and LuIII (orange), or conserved among all three viruses (gray). The remaining residues in white are identical between H-1PV and MVM only. The sialic acid binding site identified for MVM and predicted for H-1PV is delineated in purple (see bold outline). Important surface exposed residues within VR5 and VR4b are delineated in black. Icosahedral axes are indicated by the filled oval (2-fold), triangle (3-fold), and pentagon (5-fold).

**Figure 7:**
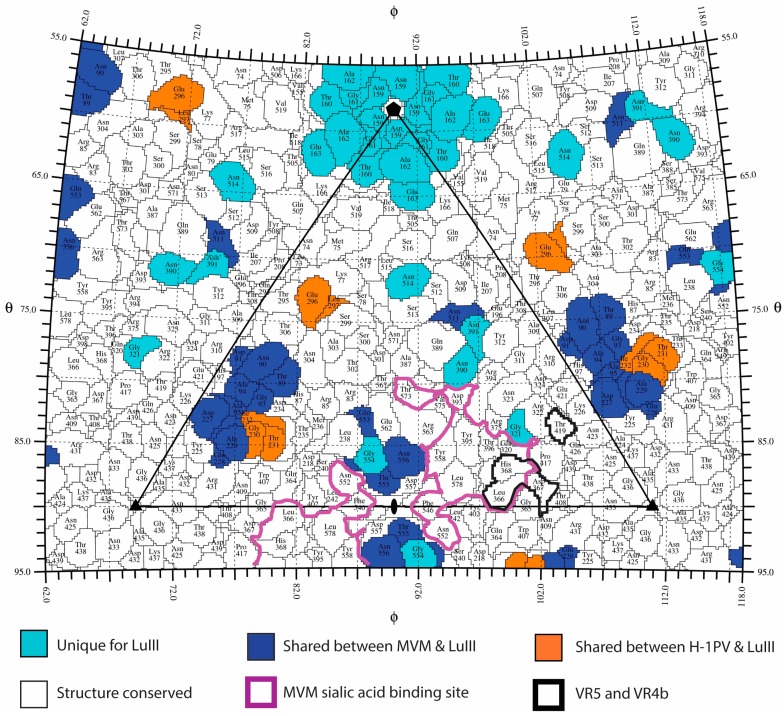
Structural conservation among the rodent protoparvoviruses. 2D projection roadmap of the LuIII capsid surface showing the viral asymmetric unit (black triangle). Colors are as indicated in the panel at the bottom. For VP2 positions in cyan, the LuIII Cα backbone deviates from MVM and H-1PV by more than 2.0 Å. Within the “shared” positions, the Cα distances between superposed VP2 structures vary by less 2.0 Å (see blue for MVM/LuIII or orange for H-1PV/LuIII). Residues in white are structurally conserved among all three viruses. The sialic acid binding site identified for MVM and predicted for H-1PV is delineated in purple. Important surface exposed residues within VR5 and VR4b are delineated in black. Icosahedral axes are indicated by the filled oval (2-fold), triangle (3-fold), and pentagon (5-fold).

Previous comparative tissue tropism studies for two strains of MVM, the prototype (MVMp) and immunosuppressive (MVMi) strains, showed that restriction occurs post cell binding and entry, but prior to genome transcription and replication [64,65], and similar observations have been made for LuIII [7,24]. However, capsid surface residues dictate MVM and H-1PV cellular entry and tissue tropism [55,57,66]. Recognition of glycans containing terminal sialic acid (SIA) is proposed to play a role in MVM tumor tropism by capsid surface residues that bind to the sLeX motif upregulated in tumor cells [29,67]. Thus, to locate potential surface residues involved in tumor tropism for the three rodent parvoviruses, VRs that differ the least among them, ≤2.0 Å in Cα position and conserved amino acid sequences, compared to CPV, FPV, and PPV, were identified. Variable regions 5 (VP2 residues 359–367) and VR4b (VP2 residues 419 and 420) satisfy the VR criteria in both H-1PV and MVM. A VP2 sequence alignment indicates that regions of sequence identity are scattered throughout the surface of the capsid, but mostly absent at the 3-fold protrusions (Figure 6). Significantly, residues in VR5 and VR4b are structurally adjacent (Figure 7), and flank the depression at the 2-fold axis where residues forming a SIA glycan receptor attachment pocket in H-1PV and MVM have been reported [57,66]. This includes I362 (equivalent to I362/I368 in MVMp/H-1PV) and H368 (equivalent to K368/H374 in MVMp/H-1PV), confirmed to dictate SIA binding by H-1PV and MVM [57,66,67], and tissue tropism for MVM [55,68]. Thus, these two VRs, located in structurally “conserved” regions of the capsid, along with conserved residues in the depression at the 2-fold axis of the capsid that dictate SIA recognition (delineated in purple, Figure 6 and Figure 7), likely dictate ability to recognize the sLeX motif to confer tumor tropism.

Towards delineating the VP2 determinant of the difference in tumor cell killing efficiency by the rodent protoparvoviruses, the remaining VRs with regions of Cα distances ≥2.0 Å were compared to identify those that vary the most in LuIII. As shown in Table 3, the entire stretch of VR residues were very similar when the rodent viruses alone are compared, but sub-regions within these are disparate (differ by >2.0 Å, see residues in parenthesis in Table 3, Figure 4). This analysis and superposition of the VP2 structures identified unique LuIII VP2 topology within VR1 (159–162), VR4a (320), VR6 (390, 391), VR7 (511), and VR8 (553–557) (Table 3, Figure 7). Residues in VR8 contribute to the unique 2-fold depression topology of LuIII surrounding the SIA binding pocket of MVM, and the predicted SIA pocket for H-1PV described above. Equivalent residues within or adjacent to these VRs dictate MVM tissue tropism, including Q320 (VR4a), which is structurally equivalent to G/E321 for MVM [28,69,70,71]. Residues in VR6 and VR7 form the wall between the depressions at the 2- and 5-fold axes (2/5-fold wall). A role for residues in the 2/5-fold wall in receptor attachment has been described for CPV [58,59,60]. Importantly, a role for 2-fold residues in altering capsid trafficking and transcription has been described for dependoparvoviruses [72,73]. Thus, the hypothesis is that the 2-fold and its surrounding residues control rodent protoparvovirus tumor tropism, as well as cell killing efficiency. These residues could cause infectivity differences by altering post-entry events preceding initial cellular receptor attachment, including endo/lysosomal trafficking and genome transcription and/or replication to determine virus specific phenotypes. A role for residues at or surrounding the 5-fold axes (VR1 (DE loop) and VR7 (HI loop)) in tropism determination has not been reported. However, the differences at the 5-fold channel may contribute to a difference in the efficiency of VP1u externalization and PLA2 function between the viruses. For example, the wider LuIII 5-fold channel may facilitate faster exit from the endo/lysosomal pathway. Overall, this comparative analysis thus identifies residues surrounding the icosahedral 2- and 5-fold axes as potential contributors to rodent protoparvovirus cell killing properties, and specific residues (Figure 7) that can be tested to pinpoint LuIII’s enhanced tumor cell killing phenotype.

## 4. Conclusions

The structure of LuIII has been determined to atomic resolution, using cryo-reconstruction to enable visualization of the individual VP2 amino acids. Comparisons of protoparvoviruses revealed conserved VP and capsid features at the family and genus levels as well as features specific to LuIII. This includes a wider and deeper 2-fold depression, a narrower 3-fold protrusion, and an open 5-fold channel. Importantly, the capsid surface acts as the first point of contact between incoming virion and cancer cells, thus directing tumor tropism. For the oncolytic rodent parvoviruses, conservation of otherwise structurally diverse VRs surrounding the depression at the 2-fold axis is observed. This 2-fold region, utilized for sialylated glycan interaction by MVM, including an sLeX tumor marker motif, is predicted to serve the same role for LuIII and H-1PV. The observation of unique sequences and structure that flank this binding site serves as a potential explanation for observed differences in tumor cell killing for LuIII. Five-fold region differences may serve to alter the VP1u externalization phenotype that could also control infectivity rates. Future studies will probe the exact role of the VP2 sequences identified in glycan usage and tumor cell killing towards improvements in tumor targeting.

## Figures and Tables

**Table 1:** Data collection, image pre-processing, and PHENIX refinement.

Parameter	LuIII
Total No. of micrographs	722
Defocus range (μm)	0.04–4.39
Electron dose (e^−^/Å^2^)	75
No. of frames/micrograph	50
Pixel size (Å/pixel)	1.064
Starting No. of particles	20,142
No. of particles used for final map	18,134
Inverse B-factor used for final map (Å^2^)	50
Resolution of final map (Å)	3.17
Residue range	37–587
Map CC	0.88
RMSD (Å)	
Bonds	0.0
Angles	0.8
All-atom clash score	11.8
Ramachandran Plot (%)	
Favored	97.8
Allowed	2.0
Outliers	0.2
Rotamer outliers (%)	0.2
No. of Cβ deviations	0.0

**Table 2:** Secondary Structure Assignments for LuIII VP2.

Capsid Core		Non-Core β-Strands	
Residue Range *	Secondary Structure	Residue Range ^#^	VP Location
125–133	α-A	82–86	within BC loop
51–54	β-A	105–109	within BC loop
		211–213	within EF loop
61–74	β-B		
521–535	β-I	156–158	within DE loop
137–147	β-D	165–167	within DE loop
268–272	β-G		
		374–378	within GH loop
112–115	β-C	393–396	within GH loop
496–500	β-H		
176–179	β-E	345–346	within GH loop
255–258	β-F	353–354	within GH loop

* Residues in core secondary structure elements (as labeled); ^#^ β-strand residues in non-core VP2 regions.

**Table 3:** Superposition Comparison of LuIII to other Protoparvoviruses.

VR	Residue Range	RMSD (Å)				
LuIII	LuIII VP2	MVM	H–1	CPV	FPV	PPV
**VR0**	90(*90–91*)91	0.3–0.8	3.5–**5.7**	0.4–1.8	0.4–1.1	0.7–1.8
**VR0**	92(*none*)94	0.6–1.2	0.6–1.1	1.3–1.9	1.0–1.6	2.1–2.7
**VR1**	159(*159–162*)162	2.0–**7.3**	1.4–2.4	1.8–3.5	2.5–3.4	1.6–3.8
**VR2**	227(*227–230*)230	0.8–3.1	2.1–3.5	1.7–1.8	0.6–3.0	1.5–2.2
**VR2**	232(*232–233*)235	0.3–2.8	0.5–1.2	2.0–3.0	1.9–3.2	0.4–1.2
**VR2**	237(*none*)241	0.3–1.0	0.2–0.7	1.8–3.2	1.7–3.1	2.0–5.0
**VR3**	296(*296–297*)298	0.7–2.7	0.5–0.8 *	1.4–3.3	1.7–3.8	2.2–4.1
**VR3**	302(*none*)303	0.1–0.2	0.4	1.5–4.5	1.5–4.5	3.2–**5.7**
**VR4a**	310(*none*)312	0.5–0.6	0.3–0.5	2.5–3.4	1.7–2.4	0.5–1.2
**VR4a**	320(*320*)322	0.5–2.8	1.0–1.9	2.3–2.8	1.7–2.4	1.3–3.9
**VR5**	359(*none*)367	0.3–1.2	0.2–1.6	2.0–**6.8**	0.9–**6.0**	0.6–1.6
**VR5**	370(*none*)371	0.4–1.2	0.4–0.6	3.5–3.7	0.1–1.0	0.8–1.0
**VR6**	386(*390–391*)392	0.7–**4.7**	0.6–3.2	2.7–4.6	0.9–4.5	1.3–3.5
**VR4b**	419(*none*)420	0.5–0.8	1.1–1.3	2.0–2.6	1.9–2.3	1.0–1.5
**VR7**	511(*511*)512	0.8–0.9	1.9–2.0	1.8–2.8	2.2	1.4–1.5
**VR8**	553(*553–557*)559	0.3–2.6	0.3–**4.4**	3.3–**5.8**	3.3–**5.8**	3.1–**6.1**
	Overall RMSD (LuIII) ^#^	0.7	0.7	1	1	1
	% VP2 Identity (LuIII) ^$^	73	70	53	52	52

RMSD = root mean squared deviation for Cα distances compared to LuIII for superposed protoparvovirus VP2 structures, the two highest RMSD values within each column are in bold; Residue Range = The range of LuIII residues within previously defined VRs. The VRs contain >2.0 Å Cα distances for 2 or more adjacent amino acids between superposed protoparvovirus structures; (*parenthesis*) = residues with Cα deviation >2.0 Å among the rodent protoparvoviruses, * indicates Cα distances of <2.0 Å in either H-1PV or MVM for residues within the parenthesis; ^#^ and ^$^ = Overall RMSD and % identity compared to LuIII VP2, respectively.

## References

[1] Bell J., McFadden G. (2014). Viruses for tumor therapy. Cell Host Microbe.

[2] Russell S.J., Peng K.W., Bell J.C. (2012). Oncolytic virotherapy. Nat. Biotechnol..

[3] Pol J., Buque A., Aranda F., Bloy N., Cremer I., Eggermont A., Erbs P., Fucikova J., Galon J., Limacher J.M. (2016). Trial watch-oncolytic viruses and cancer therapy. Oncoimmunology.

[4] Greig S.L. (2016). Talimogene laherparepvec: First global approval. Drugs.

[5] Rommelaere J., Geletneky K., Angelova A.L., Daeffler L., Dinsart C., Kiprianova I., Schlehofer J.R., Raykov Z. (2010). Oncolytic parvoviruses as cancer therapeutics. Cytokine Growth Factor Rev..

[6] Marchini A., Bonifati S., Scott E.M., Angelova A.L., Rommelaere J. (2015). Oncolytic parvoviruses: From basic virology to clinical applications. Virol. J..

[7] Paglino J., Tattersall P. (2011). The parvoviral capsid controls an intracellular phase of infection essential for efficient killing of stepwise-transformed human fibroblasts. Virology.

[8] Guetta E., Graziani Y., Tal J. (1986). Suppression of ehrlich ascites tumors in mice by minute virus of mice. J. Natl. Cancer Inst..

[9] Geletneky K., Kiprianova I., Ayache A., Koch R., Herrero Y.C.M., Deleu L., Sommer C., Thomas N., Rommelaere J., Schlehofer J.R. (2010). Regression of advanced rat and human gliomas by local or systemic treatment with oncolytic parvovirus H-1 in rat models. Neuro-Oncology.

[10] Geletneky K., Nüesch J.P., Angelova A., Kiprianova I., Rommelaere J. (2015). Double-faceted mechanism of parvoviral oncosuppression. Curr. Opin. Virol..

[11] Geletneky K., Huesing J., Rommelaere J., Schlehofer J.R., Leuchs B., Dahm M., Krebs O., von Knebel Doeberitz M., Huber B., Hajda J. (2012). Phase I/IIa study of intratumoral/intracerebral or intravenous/intracerebral administration of parvovirus H-1 (parvoryx) in patients with progressive primary or recurrent glioblastoma multiforme: ParvOryx01 protocol. BMC Cancer.

[12] Geletneky K., Hajda J., Angelova A.L., Leuchs B., Capper D., Bartsch A.J., Neumann J.O., Schoning T., Husing J., Beelte B. (2017). Oncolytic H-1 parvovirus shows safety and signs of immunogenic activity in a first phase I/IIa glioblastoma trial. Mol. Ther..

[13] Cotmore S.F., Agbandje-McKenna M., Chiorini J.A., Mukha D.V., Pintel D.J., Qiu J., Soderlund-Venermo M., Tattersall P., Tijssen P., Gatherer D. (2014). The family parvoviridae. Arch. Virol..

[14] Cotmore S.F., Tattersall P. (2006). Structure and organization of the viral genome. Parvoviruses.

[15] Richards R., Linser P., Armentrout R.W. (1977). Kinetics of assembly of a parvovirus, minute virus of mice, in synchronized rat brain cells. J. Virol..

[16] Cotmore S.F., Tattersall P. (2013). Parvoviruses: Small does not mean simple. Annu. Rev. Virol..

[17] Angelova A.L., Geletneky K., Nuesch J.P., Rommelaere J. (2015). Tumor selectivity of oncolytic parvoviruses: From in vitro and animal models to cancer patients. Front. Bioeng. Biotechnol..

[18] Bar S., Rommelaere J., Nuesch J.P. (2015). Pkη/Rdx-driven phosphorylation of PDK1: A novel mechanism promoting cancer cell survival and permissiveness for parvovirus-induced lysis. PLoS Pathog..

[19] Zádori Z., Szelei J., Lacoste M.C., Li Y., Gariépy S., Raymond P., Allaire M., Nabi I.R., Tijssen P. (2001). A viral phospholipase A2 is required for parvovirus infectivity. Dev. Cell.

[20] Tattersall P., Shatkin A.J., Ward D.C. (1977). Sequence homology between the structural polypeptides of minute virus of mice. J. Mol. Biol..

[21] Cotmore S.F., Tattersall P. (1987). The autonomously replicating parvoviruses of vertebrates. Adv. Virus Res..

[22] Cotmore S.F., D’Abramo A.M., Ticknor C.M., Tattersall P. (1999). Controlled conformational transitions in the mvm virion expose the vp1 n-terminus and viral genome without particle disassembly. Virology.

[23] Maroto B., Ramirez J.C., Almendral J.M. (2000). Phosphorylation status of the parvovirus minute virus of mice particle: Mapping and biological relevance of the major phosphorylation sites. J. Virol..

[24] Vollmers E.M., Tattersall P. (2013). Distinct host cell fates for human malignant melanoma targeted by oncolytic rodent parvoviruses. Virology.

[25] Paglino J.C., Ozduman K., van den Pol A.N. (2012). Luiii parvovirus selectively and efficiently targets, replicates in, and kills human glioma cells. J. Virol..

[26] Cho I.R., Kaowinn S., Song J., Kim S., Koh S.S., Kang H.Y., Ha N.C., Lee K.H., Jun H.S., Chung Y.H. (2015). VP2 capsid domain of the H-1 parvovirus determines susceptibility of human cancer cells to H-1 viral infection. Cancer Gene Ther..

[27] Hernando E., Llamas-Saiz A.L., Foces-Foces C., McKenna R., Portman I., Agbandje-McKenna M., Almendral J.M. (2000). Biochemical and physical characterization of parvovirus minute virus of mice virus-like particles. Virology.

[28] Kontou M., Govindasamy L., Nam H.J., Bryant N., Llamas-Saiz A.L., Foces-Foces C., Hernando E., Rubio M.P., McKenna R., Almendral J.M. (2005). Structural determinants of tissue tropism and in vivo pathogenicity for the parvovirus minute virus of mice. J. Virol..

[29] Halder S., Cotmore S., Heimburg-Molinaro J., Smith D.F., Cummings R.D., Chen X., Trollope A.J., North S.J., Haslam S.M., Dell A. (2014). Profiling of glycan receptors for minute virus of mice in permissive cell lines towards understanding the mechanism of cell recognition. PLoS ONE.

[30] Halder S., Nam H.-J., Govindasamy L., Vogel M., Dinsart C., Salomé N., McKenna R., Agbandje-McKenna M. (2013). Structural characterization of H-1 parvovirus: Comparison of infectious virions to empty capsids. J. Virol..

[31] Kailasan S., Garrison J., Ilyas M., Chipman P., McKenna R., Kantola K., Soderlund-Venermo M., Kucinskaite-Kodze I., Zvirbliene A., Agbandje-McKenna M. (2016). Mapping antigenic epitopes on the human bocavirus capsid. J. Virol..

[32] Potter C.S., Chu H., Frey B., Green C., Kisseberth N., Madden T.J., Miller K.L., Nahrstedt K., Pulokas J., Reilein A. (1999). Leginon: A system for fully automated acquisition of 1000 electron micrographs a day. Ultramicroscopy.

[33] Zheng S.Q., Palovcak E., Armache J.P., Verba K.A., Cheng Y., Agard D.A. (2017). Motioncor2: Anisotropic correction of beam-induced motion for improved cryo-electron microscopy. Nat. Methods.

[34] Rohou A., Grigorieff N. (2015). CTFFIND4: Fast and accurate defocus estimation from electron micrographs. J. Struct. Biol..

[35] Yan X., Sinkovits R.S., Baker T.S. (2007). AUTO3DEM—An automated and high throughput program for image reconstruction of icosahedral particles. J. Struct. Biol..

[36] Emsley P., Cowtan K. (2004). Coot: Model-building tools for molecular graphics. Acta Crystallogr. Sect. D Biol. Crystallogr..

[37] Pettersen E.F., Goddard T.D., Huang C.C., Couch G.S., Greenblatt D.M., Meng E.C., Ferrin T.E. (2004). UCSF Chimera—A visualization system for exploratory research and analysis. J. Comput. Chem..

[38] Bordoli L., Kiefer F., Arnold K., Benkert P., Battey J., Schwede T. (2008). Protein structure homology modeling using SWISS-MODEL workspace. Nat. Protoc..

[39] Diffoot N., Chen K.C., Bates R.C., Lederman M. (1993). The complete nucleotide sequence of parvovirus luiii and localization of a unique sequence possibly responsible for its encapsidation pattern. Virology.

[40] Carrillo-Tripp M., Shepherd C.M., Borelli I.A., Venkataraman S., Lander G., Natarajan P., Johnson J.E., Brooks C.L., Reddy V.S. (2009). VIPERdb2: An enhanced and web api enabled relational database for structural virology. Nucleic Acids Res..

[41] Tang G., Peng L., Baldwin P.R., Mann D.S., Jiang W., Rees I., Ludtke S.J. (2007). EMAN2: An extensible image processing suite for electron microscopy. J. Struct. Biol..

[42] Kleywegt G.J., Jones T.A. (1996). XdlMAPMAN and xdlDATAMAN—Programs for reformatting, analysis and manipulation of biomacromolecular electron-density maps and reflection data sets. Acta Crystallogr. Sect. D Biol. Crystallogr..

[43] Adams P.D., Afonine P.V., Bunkoczi G., Chen V.B., Davis I.W., Echols N., Headd J.J., Hung L.W., Kapral G.J., Grosse-Kunstleve R.W. (2010). Phenix: A comprehensive python-based system for macromolecular structure solution. Acta Crystallogr. Sect. D Biol. Crystallogr..

[44] Chen V.B., Arendall W.B., Headd J.J., Keedy D.A., Immormino R.M., Kapral G.J., Murray L.W., Richardson J.S., Richardson D.C. (2010). Molprobity: All-atom structure validation for macromolecular crystallography. Acta Crystallogr. Sect. D Biol. Crystallogr..

[45] Krissinel E., Henrick K. (2004). Secondary-structure matching (SSM), a new tool for fast protein structure alignment in three dimensions. Acta Crystallogr. Sect. D Biol. Crystallogr..

[46] Wu H., Rossmann M.G. (1993). The canine parvovirus empty capsid structure. J. Mol. Biol..

[47] Simpson A.A., Hebert B., Sullivan G.M., Parrish C.R., Zadori Z., Tijssen P., Rossmann M.G. (2002). The structure of porcine parvovirus: Comparison with related viruses. J. Mol. Biol..

[48] Simpson A.A., Chandrasekar V., Hebert B., Sullivan G.M., Rossmann M.G., Parrish C.R. (2000). Host range and variability of calcium binding by surface loops in the capsids of canine and feline parvoviruses. J. Mol. Biol..

[49] Xiao C., Rossmann M.G. (2007). Interpretation of electron density with stereographic roadmap projections. J. Struct. Biol..

[50] Castellanos M., Perez R., Rodriguez-Huete A., Grueso E., Almendral J.M., Mateu M.G. (2013). A slender tract of glycine residues is required for translocation of the VP2 protein n-terminal domain through the parvovirus mvm capsid channel to initiate infection. Biochem. J..

[51] Halder S., Ng R., Agbandje-McKenna M. (2012). Parvoviruses: Structure and infection. Future Virol..

[52] Hafenstein S., Bowman V.D., Sun T., Nelson C.D., Palermo L.M., Chipman P.R., Battisti A.J., Parrish C.R., Rossmann M.G. (2009). Structural comparison of different antibodies interacting with parvovirus capsids. J. Virol..

[53] Kaufmann B., Lopez-Bueno A., Mateu M.G., Chipman P.R., Nelson C.D., Parrish C.R., Almendral J.M., Rossmann M.G. (2007). Minute virus of mice, a parvovirus, in complex with the fab fragment of a neutralizing monoclonal antibody. J. Virol..

[54] Wikoff W.R., Wang G., Parrish C.R., Cheng R.H., Strassheim M.L., Baker T.S., Rossmann M.G. (1994). The structure of a neutralized virus: Canine parvovirus complexed with neutralizing antibody fragment. Structure.

[55] Lopez-Bueno A., Segovia J.C., Bueren J.A., O’Sullivan M.G., Wang F., Tattersall P., Almendral J.M. (2008). Evolution to pathogenicity of the parvovirus minute virus of mice in immunodeficient mice involves genetic heterogeneity at the capsid domain that determines tropism. J. Virol..

[56] Barbis D.P., Chang S.F., Parrish C.R. (1992). Mutations adjacent to the dimple of the canine parvovirus capsid structure affect sialic acid binding. Virology.

[57] Lopez-Bueno A., Rubio M.P., Bryant N., McKenna R., Agbandje-McKenna M., Almendral J.M. (2006). Host-selected amino acid changes at the sialic acid binding pocket of the parvovirus capsid modulate cell binding affinity and determine virulence. J. Virol..

[58] Hueffer K., Govindasamy L., Agbandje-McKenna M., Parrish C.R. (2003). Combinations of two capsid regions controlling canine host range determine canine transferrin receptor binding by canine and feline parvoviruses. J. Virol..

[59] Govindasamy L., Hueffer K., Parrish C.R., Agbandje-McKenna M. (2003). Structures of host range-controlling regions of the capsids of canine and feline parvoviruses and mutants. J. Virol..

[60] Hafenstein S., Palermo L.M., Kostyuchenko V.A., Xiao C., Morais M.C., Nelson C.D., Bowman V.D., Battisti A.J., Chipman P.R., Parrish C.R. (2007). Asymmetric binding of transferrin receptor to parvovirus capsids. Proc. Natl. Acad. Sci. USA.

[61] DiPrimio N., Asokan A., Govindasamy L., Agbandje-McKenna M., Samulski R.J. (2008). Surface loop dynamics in adeno-associated virus capsid assembly. J. Virol..

[62] Farr G.A., Tattersall P. (2004). A conserved leucine that constricts the pore through the capsid fivefold cylinder plays a central role in parvoviral infection. Virology.

[63] Bleker S., Sonntag F., Kleinschmidt J.A. (2005). Mutational analysis of narrow pores at the fivefold symmetry axes of adeno-associated virus type 2 capsids reveals a dual role in genome packaging and activation of phospholipase A2 activity. J. Virol..

[64] Spalholz B.A., Tattersall P. (1983). Interaction of minute virus of mice with differentiated cells: Strain-dependent target cell specificity is mediated by intracellular factors. J. Virol..

[65] Previsani N., Fontana S., Hirt B., Beard P. (1997). Growth of the parvovirus minute virus of mice mvmp3 in el4 lymphocytes is restricted after cell entry and before viral dna amplification: Cell-specific differences in virus uncoating in vitro. J. Virol..

[66] Allaume X., El-Andaloussi N., Leuchs B., Bonifati S., Kulkarni A., Marttila T., Kaufmann J.K., Nettelbeck D.M., Kleinschmidt J., Rommelaere J. (2012). Retargeting of rat parvovirus h-1pv to cancer cells through genetic engineering of the viral capsid. J. Virol..

[67] Nam H.J., Gurda-Whitaker B., Gan W.Y., Ilaria S., McKenna R., Mehta P., Alvarez R.A., Agbandje-McKenna M. (2006). Identification of the sialic acid structures recognized by minute virus of mice and the role of binding affinity in virulence adaptation. J. Biol. Chem..

[68] Rubio M.P., Lopez-Bueno A., Almendral J.M. (2005). Virulent variants emerging in mice infected with the apathogenic prototype strain of the parvovirus minute virus of mice exhibit a capsid with low avidity for a primary receptor. J. Virol..

[69] Agbandje-McKenna M., Llamas-Saiz A.L., Wang F., Tattersall P., Rossmann M.G. (1998). Functional implications of the structure of the murine parvovirus, minute virus of mice. Structure.

[70] Ball-Goodrich L.J., Tattersall P. (1992). Two amino acid substitutions within the capsid are coordinately required for acquisition of fibrotropism by the lymphotropic strain of minute virus of mice. J. Virol..

[71] Ball-Goodrich L.J., Moir R.D., Tattersall P. (1991). Parvoviral target cell specificity: Acquisition of fibrotropism by a mutant of the lymphotropic strain of minute virus of mice involves multiple amino acid substitutions within the capsid. Virology.

[72] Aydemir F., Salganik M., Resztak J., Singh J., Bennett A., Agbandje-McKenna M., Muzyczka N. (2016). Mutants at the 2-fold interface of adeno-associated virus type 2 (AAV2) structural proteins suggest a role in viral transcription for aav capsids. J. Virol..

[73] Salganik M., Aydemir F., Nam H.J., McKenna R., Agbandje-McKenna M., Muzyczka N. (2014). Adeno-associated virus capsid proteins may play a role in transcription and second-strand synthesis of recombinant genomes. J. Virol..

